# P026. Pilot study on the use of coenzyme Q10 in a group of patients with episodic migraine without aura

**DOI:** 10.1186/1129-2377-16-S1-A186

**Published:** 2015-09-28

**Authors:** Giorgio Dalla Volta, Daniela Carli, Paola Zavarise, Gaelle Ngonga, Stefano Vollaro

**Affiliations:** Brescia Headache Centre, Istituto Clinico Città di Brescia, Brescia, Italy

## Introduction

Coenzyme Q10 is a naturally occurring substance and essential element of the mitochondrial electron transport chain. There has been a recent interest in the role that mitochondria may play in migraine pathogenesis. If indeed migraine results from mitochondrial dysfunction, then coenzyme Q10 could be used as a successful migraine preventive therapy. Abnormal mitochondrial function translates into high intracellular penetration of Ca(2+), excessive production of free radicals, and deficient oxidative phosphorylation, which ultimately causes energy failure in neurons and astrocytes, thus triggering migraine mechanisms. The objective of this investigation was to confirm the efficacy of coenzyme Q10 as a preventive treatment for migraine headaches.

## Materials and methods

We selected from the Headache Center of the “Istituto Clinico Città di Brescia” a population of 40 patients aged between 18 and 65 years, suffering from migraine without aura with a headache frequency between 3 to 6 crises/month (4 to 12 days with headache/month), not assuming other migraine preventive therapy. They were randomly assigned to treatment with coenzyme Q10 300 mg (20 patients) or 600 mg (20 patients) once a day for 3 months. Patients were evaluated for frequency, duration, intensity of pain, response to trigger factors and response to their habitual analgesic drug (data obtained from the diary of headache delivered at first visit). The improvement of the thermographic pattern was also evaluated.

## Results

A reduction in frequency and intensity of more than 50% in 25 patients, 10 patients reported reduction of more than 50% in the intensity but not in frequency (<50%), 5 patients had no improvement of symptoms. In all patients the complexity of the accompanying symptoms were less severe and they noted a reduction of the response to trigger factors.

## Discussion and conclusions

Coenzyme Q10 is an essential element of the mitochondrial electron transport chain and has been shown to improve mitochondrial oxidative phosphorylation in humans. Coenzyme Q10, which can be administered orally with an excellent side-effect profile, appears to be a good migraine preventive.

Written informed consent to publication was obtained from the patient(s).

